# Personalized Virtual Reality Human-Computer Interaction for Psychiatric and Neurological Illnesses: A Dynamically Adaptive Virtual Reality Environment That Changes According to Real-Time Feedback From Electrophysiological Signal Responses

**DOI:** 10.3389/fnhum.2021.596980

**Published:** 2021-02-12

**Authors:** Iakovos Kritikos, Georgios Alevizopoulos, Dimitris Koutsouris

**Affiliations:** ^1^Department of Bioengineering, Imperial College London, South Kensington Campus, London, United Kingdom; ^2^Psychiatric Clinic, Agioi Anargyroi General Oncological Hospital of Kifisia, Athens, Greece; ^3^Biomedical Engineering Laboratory, School of Electrical and Computer Engineering, National Technical University of Athens, Athens, Greece

**Keywords:** mental illnesses, neurological illnesses, electrophysiology, noninvasive device, electrodermal activity sensor, virtual reality, human-computer interaction, real time adaptation

## Abstract

Virtual reality (VR) constitutes an alternative, effective, and increasingly utilized treatment option for people suffering from psychiatric and neurological illnesses. However, the currently available VR simulations provide a predetermined simulative framework that does not take into account the unique personality traits of each individual; this could result in inaccurate, extreme, or unpredictable responses driven by patients who may be overly exposed and in an abrupt manner to the predetermined stimuli, or result in indifferent, almost non-existing, reactions when the stimuli do not affect the patients adequately and thus stronger stimuli are recommended. In this study, we present a VR system that can recognize the individual differences and readjust the VR scenarios during the simulation according to the treatment aims. To investigate and present this dynamically adaptive VR system we employ an Anxiety Disorder condition as a case study, namely arachnophobia. This system consists of distinct anxiety states, aiming to dynamically modify the VR environment in such a way that it can keep the individual within a controlled, and appropriate for the therapy needs, anxiety state, which will be called “desired states” for the study. This happens by adjusting the VR stimulus, in real-time, according to the electrophysiological responses of each individual. These electrophysiological responses are collected by an external electrodermal activity biosensor that serves as a tracker of physiological changes. Thirty-six diagnosed arachnophobic individuals participated in a one-session trial. Participants were divided into two groups, the Experimental Group which was exposed to the proposed real-time adaptive virtual simulation, and the Control Group which was exposed to a pre-recorded static virtual simulation as proposed in the literature. These results demonstrate the proposed system’s ability to continuously construct an updated and adapted virtual environment that keeps the users within the appropriately chosen state (higher or lower intensity) for approximately twice the time compared to the pre-recorded static virtual simulation. Thus, such a system can increase the efficiency of VR stimulations for the treatment of central nervous system dysfunctions, as it provides numerically more controlled sessions without unexpected variations.

## Introduction

Over the past several years, virtual reality (VR) has undergone considerable progress and has been proven as a useful tool to improve many psychiatric and neurological treatments, like those implemented for Depression (Falconer et al., 2016; Lindner et al., 2019; Schleider et al., 2019), Social Anxiety (Kampmann et al., 2016; Kim et al., 2017; Chesham et al., 2018), Post-Traumatic Stress Disorder (Beidel et al., 2019; Kothgassner et al., 2019; Loucks et al., 2019), Schizophrenia (Ruse et al., 2014; Spanlang et al., 2019; Gainsford et al., 2020), Alcohol/Drug Addiction (Bordnick and Washburn, 2019; Ghiţă et al., 2019; Segawa et al., 2020), Alzheimer’s disease (Serino et al., 2017; Caggianese et al., 2018; Clay et al., 2020), Epilepsy (Maidenbaum et al., 2019; Höller et al., 2020; House et al., 2020), Stroke (Lupu et al., 2016; Gamito et al., 2017; Iruthayarajah et al., 2017; Kritikos et al., 2019b; Matamala-Gomez et al., 2020a), Autism (Newbutt et al., 2016, 2020; Meindl et al., 2019), chronic pain (Jones et al., 2016; Ahmadpour et al., 2019; Matamala-Gomez et al., 2019). VR is usually combined with invasive or non-invasive electrodes to assist each treatment accordingly (Solcà et al., 2018; Burin et al., 2020; Matamala-Gomez et al., 2020b). VR act as a driver for such disorders, stimulating appropriate emotions, memories, and physical movements that underpin the treatment procedures. However, at the moment VR simulations do not take into consideration the unique personality of each individual separately, which is a core factor that substantially affects the efficacy and duration of the treatment. Those simulations are predetermined, predesigned, provide very specific scenarios, and do not take into account the nature, the character, and the behavior of each patient. In particular, overt characteristics such as age, sex, ethnic and cultural differences (Andersson et al., 1993; Gagliese and Melzack, 2003; Brenes et al., 2008; Ochoa et al., 2012), economic, marital, and educational status differences (Yu and Williams, 1999; Robards et al., 2012), individuals with an inclination to nausea and loss of spatial awareness (Nichols and Patel, 2002; Sharples et al., 2008; Mittelstaedt et al., 2018), or more complex characteristics, such as individuals who suffer from comorbidity of psychiatric, neurological or medical conditions (Sartorious, 2013; Hesdorffer, 2016; Plana-Ripoll et al., 2019), can significantly affect the VR interventions. Thus, those personal features provoke significant outcome variations in different patients while they receive the same VR simulation treatment.

In this study, the question which we investigate is whether it is possible, to create VR scenarios that adapt during the simulation, in real-time, to each patient’s unique personality. This approach is based on the fact that every emotion, memory, and physical movement within the VR environment provokes a reaction to the sympathetic and parasympathetic nervous systems (Pugnetti et al., 1996; Schiza et al., 2019). So, by measuring this reaction continuously, during the simulation, it is possible to re-adjust the VR environment dynamically, in real-time, according to the treatment’s goal. To demonstrate this approach, and to present an initial real-time adaptive VR system, we use Arachnophobia (Spider Phobia) as a case study with measurement employing an electrodermal activity sensor (EDA). This choice was intentionally made due to the knowledge that VR dominates the device-oriented treatments for Anxiety Disorders, so there is a lot of literature on which we can rely and construct a dynamically adaptive VR system based on reliable sources. Arachnophobia was selected because spiders can be elaborated more easily as virtual objects in the virtual environment compared to other more complex scenarios. The EDA sensor was selected because it is a well-studied, not complicated, and effective one-channel electrode which is a reliable start as a means of feedback.

**Figure 1:**
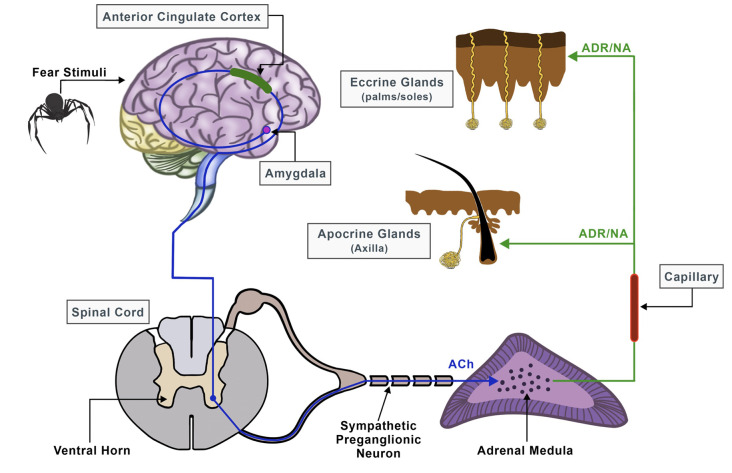
Neural pathway of sweat secretion from the brain to the sweat glands, triggered by stressful conditions.

Electrodermal activity signals are used effectively as an indicator of sympathetic skin responses (SSR) of the central and peripheral nervous system since they can help in the recognition of various emotional stages guided by changes in skin electrical properties (Christopoulos et al., 2019). The autonomic sympathetic and parasympathetic nervous systems work in an antagonistic fashion to preserve homeostasis by mediating pivotal physiological activities such as heartbeat, respiration, muscle movement, blood pressure, and secretion of sweat (Kerns et al., 2013). Particularly, EDA has played an important role in the field of psychophysiology, and thanks to its accessibility, sudomotor activity, and, thus, sweat gland secretion can be directly assessed and linked to specific cognitive and behavioral states (Vetrugno et al., 2003). There are two primary categories of sweat glands: the eccrine and apocrine sweat glands and their function is to either control the body temperature (thermoregulatory sweating) or respond to emotional, cognitive, and physiological stimuli (emotional sweating; Asahina et al., 2003; Hu et al., 2018). Emotional sweating affects mainly the palms, soles, and axillae and can occur predominantly in fear conditions and aversive situations like in cases of specific phobias and traumatic events (Harker, 2013; Wegerer et al., 2013). It follows a complex pathway that is coordinated by both, the central and autonomous nervous systems, and depends on the discharge of neurotransmitters like acetylcholine (Ach), noradrenaline (NA), and adrenaline (ADR; Harker, 2013; Hu et al., 2018; Figure 1).

Phobias are one of the classes belonging to the spectrum of Anxiety Disorders accompanied by extreme, irrational, and constant fear of specific objects and/or situations. For instance, arachnophobia is a simple phobia that is triggered by the actual physical presence or imaginary presence of spiders and other arachnids (Sarlo et al., 2002; McGuigan and Andreassi, 2006; Mulholland, 2017). It affects approximately 3.5–6.1% of the global population, with a high prevalence compared to other simple phobias (Schmitt and Müri, 2009). These types of phobias are associated with an abnormal increase of sympathetic activity as a defensive mechanism to elevated arousal and fear conditions, along with behaviors of disgust, avoidance, and escapism (Wiederhold, 2008). To address this disorder, phobic individuals are driven to the use of medications that can help reduce the heart rate and blood pressure, as well as mitigate panic attacks (Pachana et al., 2007). Besides medication, occasionally a more psychotherapeutic approach is applied, which usually relies on Exposure Therapy, a form of cognitive-behavioral therapy (CBT) treatment that aims to habituate patients with their fears and control the related anxiety (Pachana et al., 2007; Taffou et al., 2017). During Exposure Therapy, for instance, patients learn how to interact with the anxiety-triggering stimulus by being repeatedly subjected to it, either in real-life (*in vivo* Exposure Therapy) or in imaginary situations (Virtual Exposure Therapy), always in a safe setting, supervised by a clinician.

Recently, virtual reality (VR) technology has undergone considerable progress and can be a useful tool in clinical settings by assisting therapists in stimulating desirable psychological states, to create a more precise diagnosis, and, finally, proposing a more integrated treatment (Krijn et al., 2004a, b; Kim et al., 2017; Freeman et al., 2018). Previous research has already highlighted the potential of VR to excite the senses as effectively as a real-life stimulus can (Kritikos et al., 2020), by introducing Virtual Reality Exposure Therapy (VRET) as a solid procedure in the assessment of fear responses (Gamito et al., 2017; Iruthayarajah et al., 2017; House et al., 2020). Specifically, Exposure Therapy frequently benefits from VR systems to graphically reproduce vivid simulations for any kind of stress-related stimuli and induce real emotions, such as fear induced by the presence of spiders or other phobic stimuli (Bun et al., 2017). Taking into consideration the therapeutic aspect of VR environments, the latter are extensively used in studies related to the treatment of phobias and numerous other anxiety syndromes (Bosse et al., 2014; Taffou et al., 2017). VRET simulations are usually combined with physiological measurements and, thus, sympathetic activity measurements are collected to validate stress levels and classify various anxiety states (Koelstra et al., 2012; Cho et al., 2017). Therefore, this combinatorial recording procedure, along with the data compiled, can serve as key components to the diagnosis and elucidation of the severity of each disorder, as well as to the identification of the clients’ reactions and overall progress (Peperkorn et al., 2015; Diemer et al., 2016; Yeh et al., 2018).

Although a wide range of studies demonstrate that each emotional state can be distinctively mapped through its matching physiological signals, emotions are unique and individually expressed by different personalities (Lisetti and Nasoz, 2004; Kritikos et al., 2019a). Also, emotional responses are multidimensional since they rely on cognitive, mental, and somatic perspectives and cannot be adequately defined by a valid theory (Newbutt et al., 2016). People encounter different experiences and memories throughout their lives, react differently to known or unknown stimuli and thus, consciously or unconsciously, perceive and interpret a stressor in their individual and unique way (Newbutt et al., 2020). The use of VR when the need arises to create an environment of fear for therapy reasons is well-established (Koelstra et al., 2012). However, current VR systems do not prioritize this diversity, and yield a uniform and common approach toward all participants that is based on an immersion/presence-specific setting, yet lacks personalized information for each case (Wilson and Soranzo, 2015). Admittedly, several researchers attempted to create more tailored VR systems to improve treatment outcomes (Ćosić et al., 2010; Bermudez et al., 2019; Heyse et al., 2019a, b; Lin et al., 2019; Kritikos et al., 2019c). However, there is no explicit investigation so far that highlights differences in the patient’s behavior during a real-time adaptive VR treatment and a predefined VR treatment.

In this study, we propose a VR system implementation for which our hypothesis is whether this system can dynamically adapt during the session, in real-time, to each participant’s unique behavioral patterns and according to the treatment’s goals. This integrated system could be a valuable tool for clinicians, especially in terms of devising personalized treatments, based on each patient’s unique perception of their phobia. Specifically, we have designed and implemented a VR system, which we consider that constantly modifies the virtual phobic scenario of the simulation and depends on the patient’s specific emotional responses, utilizing the feedback information it receives from bio-signals in real-time. With arachnophobia as the case study, we attempted to induce a personalized stimulus that ensures a different stress reaction during the session, by altering the spiders’ appearance and action patterns according to the users’ constant electrodermal results. Bio-signals were recorded by an EDA sensor which is considered a proper method to estimate fear reaction, as it can easily detect increased anxiety reactions by simply measuring participants’ sweat secretion activity. To investigate our hypothesis and examine the efficacy of the proposed dynamically adaptive VR system, we compared it to a pre-recorded static VR system as it is currently used, in which the EDA sensor does not affect the VR environment but is used only as a measurement tool for the anxiety. Practically, we compared those systems by measuring the EDA deviation from the selected anxiety state each time. The system in which the EDA measurements have the least deviation from the selected anxiety state for longer is considered of superior efficacy.

## System Description

We designed a dynamically adaptive VR system that met our needs for this study and was sufficiently qualified with the following characteristics.

### Hardware

**Figure 2:**
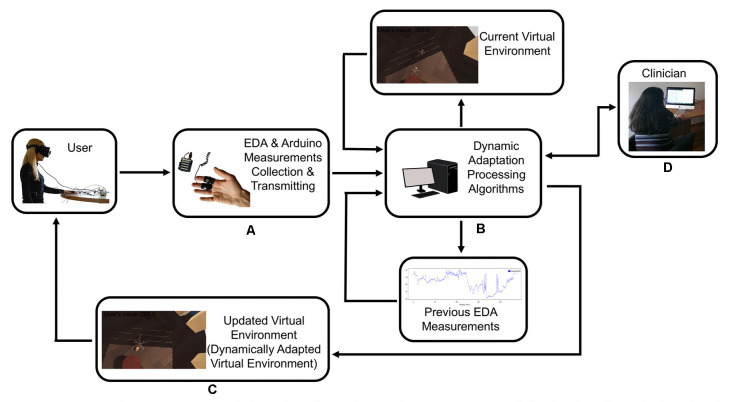
The user provides feedback to the system with their physiological response, and the system recalibrates itself to adapt the virtual scenario to the user’s response. **(A)** Electrodermal activity sensor (EDA)measurements collection and transmitting. **(B)** Dynamic adaptation processing. **(C)** Dynamically adapted virtual environment. **(D)** Clinician has the control of the system at all time.

The system included a desktop computer with the following specifications: Graphics Card: NVIDIA GeForce GTX 1070, CPU: AMD Ryzen 7 2700X, RAM: 16 GB G.Skill TridentZ DDR4, Video Output: HDMI 1.3, USB Ports: 3× USB 3.0 and 1× USB 2.0; an Oculus Rift VR Headset; and an Arduino Uno connected with a Seeed 101020052 Grove Electrodermal Activity Sensor which measures the electrical conductance of the skin (Figure 2).

### Software

The software used included the following: (a) Windows 10 Operating System with drivers for Oculus Rift installed; (b) Unity 3D, which was used as the basic program for creating the virtual environment. All hardware pieces (i.e., sensors, controllers, trackers, headset, et cetera) were controlled by Unity 3D; (c) Blender 3D Computer Graphics Software was used for creating 3D objects, animated visual effects, UV Mapping, and materials integrated with Unity 3D; (d) Adobe Photoshop was used for creating images for the materials integrated with Blender; (e) OVR Plugin was used for the operation of the Oculus Rift equipment in Unity 3D; (f) The Arduino IDE was used to develop the script that reads values from the GSR sensor which are then utilized appropriately in our software developed in Unity. The Arduino transmits the data at 100 Hz; and (g) Python was used with JupyterLab for data analysis of the data extracted from the simulation.

### System Procedure

Initially, the system was launched to record the user’s electrodermal response in resting conditions, without any stimulus introduced. After the system initialization, phobic stimuli (e.g., spiders) of graded intensity were installed, triggering different stress reactions according to each person’s perception of fear. As soon as the EDA response was recorded and processed, the system adjusted and updated the virtual scenario parameters to meet the anxiety reaction levels of the user each time. Essentially, the user provided feedback to the system with their physiological response, and the system recalibrated itself to adapt the virtual scenario to the user’s response. A detailed system description is described below:

#### Initialization

During minute 1, for the buffer to be empty, any previously stored values that the Arduino transmitted are discarded. Before the simulation procedure commences, each user can see the virtual room with no fear stimuli present; in our case, spiders. At that moment the system is initiated. The EDA sensor can make 100 measurements per second. During that first minute, the simulation does not start and the user remains in this stimuli-free virtual room until their EDA measurements reach within ±8% of the mean value of the measurements which are gathered during minute 1. This process was constructed to obtain the user’s “Relaxed EDA value,” which corresponds to their unique calm or rest state (unstressed state). This value is considered fundamental for the initialization of the system, since each person exhibits distinct initial EDA values at the beginning of their simulation, driven mainly by potential exogenous factors, which can affect their mental calmness and, thus, their sweat secretion.

#### Normalization

Once the simulation starts, every EDA measurement is normalized by dividing the incoming EDA value with the Relaxed EDA value. This normalization is implemented to define a common comparison factor for our system, bearing in mind that each user provides different EDA values. Therefore, we propose that the transformation of EDA values should fall between a range from 0 to 1. This means that a normalized value of 0.95 indicates an EDA value very close to the unstressed state value with low anxiety levels, while a normalized value of 0.45 indicates an EDA value less than half the unstressed state value, driven by the high conductivity induced by the extensive sweating response and high anxiety levels. Therefore, Low Anxiety levels, equals low conductivity, equals high resistance, equals high EDA measurements, equals high Normalized EDA measurements, and closer to 1.0. High Anxiety levels, equals high conductivity, equals low resistance, equals low EDA measurements, equals low Normalized EDA measurements, and closer to 0.0. So, for this research, when we mention **Low Anxiety** this corresponds to **High Normalized EDA values** while **High Anxiety** corresponds to **Low Normalized EDA values**.

#### States

Dynamic adaptation is based on the ability of the system to generate appropriate anxiety-inducing stimuli to bring each user’s sweat secretion—and, therefore, the intensity of their anxiety—close to certain states. Each state is defined as a specific, pre-selected range of EDA values. For the aforementioned purposes we have selected the following three states:

• **Rest State (State-0)**: in this state, no anxiety-inducing stimuli appear in the VR environment, the user is not expected to experience any simulation-related anxiety. Therefore, the Normalized EDA values are expected to be high and within the range of 1.0–0.92. This state is displayed at the beginning of each simulation to determine the user’s Relaxed EDA value in addition to restoring the user’s anxiety levels after simulations that include anxiety-inducing stimuli.
• **Low-Intensity State (State-1)**: in this state, the system is expected to generate enough stimuli to bring and keep the user’s Normalized EDA values within the range of 0.8–0.7. When the user’s sweat secretion is within this range, their emotional state can be considered as mildly anxious.
• **High-Intensity State (State-2)**: in this state, the system is expected to generate enough stimuli to bring and keep the user’s Normalized EDA value within the range of 0.5–0.4. When the user’s sweat secretion is within this range, their emotional condition can be considered as rather anxious.

Each State’s representative value is defined by its lowest threshold (i.e., 0.92 for State-0, 0.7 for State-1, and 0.4 for State-2).

#### Stimuli Parameterization

To achieve satisfactory fluctuations as regards the intensity of the feared stimuli and, thus, reach and sustain the appropriately chosen controlled anxiety states (states desired by the respective clinician for the therapy), we have designed a virtual room where the spiders constantly appear, disappear and change in size, velocity, direction, jumping force based on specific parameterized functions. These parameters and their functions were created following the guideless from several publications that have studied VR simulation for Spider Phobias (Shiban et al., 2013, 2015a,b, 2016; Miloff et al., 2016, 2019; Lindner et al., 2017; Juvrud et al., 2018; Tardif et al., 2019). In particular, the spiders differ in terms of the following parameters:

• Stimulus Generation Frequency (SGF)
• Stimulus Jumping Force (SJF)
• Stimulus Probability Moving Towards User (SPU)
• Stimulus Size (SS)
• Stimulus Velocity (SV)

The primary variable that determines the adjustment of the selected system parameters is the Correction variable. First, the last received measurement from the EDA sensor is Normalized based on the Relaxed EDA value. Then, the Normalized EDA value is subtracted from the state’s representative value. The result is the Correction variable (Equation 1) that ranges from −0.4 to 0.6 during State-1 and from −0.7 to 0.3 during State-2. This range was defined based on system iterations. If the Correction variable is less than zero, the characteristics of the next generated spider need to provoke a stronger fear stimulus, because the user’s EDA values are lower than the desired ones, i.e., they are more scared and anxious than desired. If the Correction variable is equal to or more than zero, a weaker fear stimulus is to be introduced since the user’s EDA values are higher than the desired ones.


(1) correction=desired_eda_normalized−user_eda_normalized

(2) $$user\_eda\_normalized=\frac{user\_eda}{relaxed\_eda}$$

(3) $$desired\_eda\_normalized=\{\begin{array}{c} \text{0.92},\\ \text{0.7,}\\ \text{0.4,} \end{array}\begin{array}{c} State—\text{0}\\ State—\text{1}\\ State—\text{2} \end{array}$$

(4) user_position=(x,y,z)

Let’s assume that we are always aware of the user’s position within the virtual room (Equation 4). Each spider is then generated according to the following parameters:

##### A. Stimulus Generation Frequency (SGF)

The number of stimuli that appear simultaneously in the virtual environment changes according to a function of the Correction variable so that when the correction variable is negative, spiders are then generated more frequently (every fewer frames) and vice versa (Equation 5).


(5) $$generation\_frequency=\{{\;}_{\text{1040 }-\text{ 1200 (}correction\text{ + 0.6), }State\text{ = }High}^{\text{840 }-\text{ 1200 }\left(correction\;+\text{ 0.6}\right),\;State\;=\;Low}$$

##### B. Stimulus Jumping Force (SJF)

This parameter is defined as the force (in Newtons) with which the spider will jump on the vertical (*y*) axis. The jumping force acts as a function of the Correction variable so that when the correction variable is negative, the spider jumps higher and vice versa (Equation 6). Note that only spiders that move towards the user can jump.


(6) $$jumping\_force=-\text{0.03}+\frac{\text{0.364}}{correction+\text{4.6}}$$

##### C. Stimulus Probability Moving Towards User (SPU)

Upon generation, a spider might detour from its route towards its destination to approach the user, with a probability of* X*. This probability is a function of the Correction variable so that when the correction variable is negative, then the spider is more probable to move towards the user and vice versa (Equation 7).


(7) $$probability={\left(\text{1}-\frac{correction+\text{0.6}}{\text{1.4}}\right)}^{\text{2}}$$

##### D. Stimulus Size (SS)

The size of the generated spider changing is a function of the Correction variable so that when the correction variable is negative, the spider is generated larger and vice versa (Equation 8). The spiders scale up and down in relation to the original size given by the designer, to meet the system’s needs.


(8) $$size=\text{0.3}+\text{0.9}\times \text{0}{\text{.2}}^{correction}$$

##### E. Stimulus Velocity (SV)

The velocity with which the stimuli move towards their destination is a function of the Correction variable so that when the correction variable is negative, the spider will then move quicker and vice versa. Basically, in every frame, the spider’s velocity is pre-defined (Equation 9).


(9) $$velocity=\text{0.006}-\text{0.003}\frac{correction+\text{0.6}}{\text{1.4}}$$

**Figure 3:**
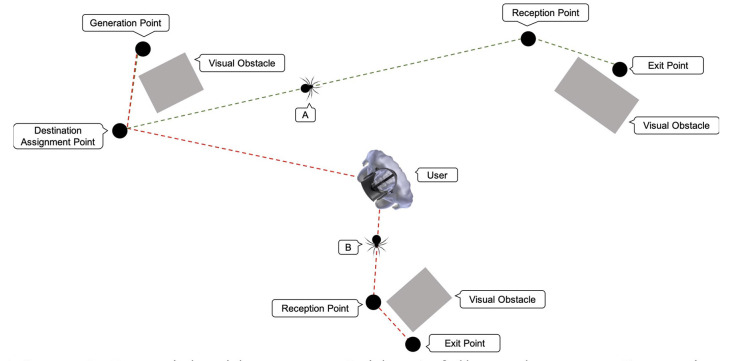
Potential spider routes: spider (A) follows the route: Generation Point, Destination Assignment Point, Reception (gathering) Point, and Exit Point. Spider (B) follows the route: Generation Point, Destination Assignment Point, User, Reception Point, and Exit Point.

**Figure 4:**
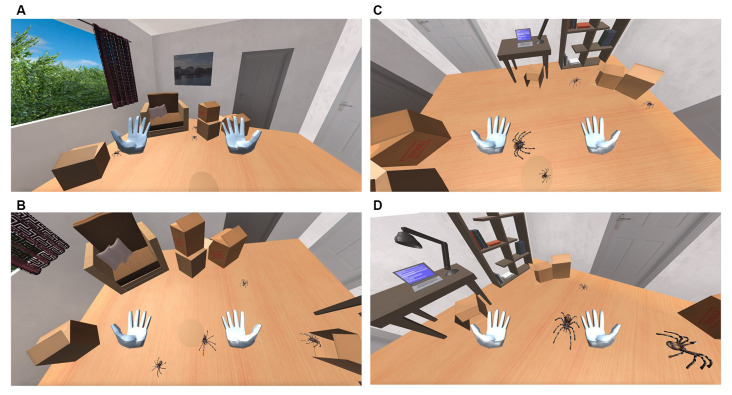
**(A)** Spiders appears behind the boxes. **(B)** Spiders moving towards the user. **(C)** Spiders are jumping. **(D)** Spider size increases.

As shown in (Figure 3), spiders follow the following pathway: Each spider pops up in a generation point; which is a point in the virtual room from where spiders are generated. There are four-generation points in the room. The generation point of each spider is randomly defined. An object (e.g., a box) is placed in front of each generation point (Figure 4A) so that the emerging spider will not be detectable by the user. Eventually, the user becomes aware of each generated spider appearing from behind the object suddenly causing him alarm and thus making the process as realistic as possible. Once a spider is generated and appears in the user’s field of view, it starts moving towards a destination assignment point (Figure 4B). The destination assignment points have a fixed location and are close to each generation point. Each spider must move toward the destination assignment point so that its respective destination will be determined. Also, these points serve an additional role by ensuring that spiders do not collide with each other or other objects while setting out for their final destination.

The spiders’ destination can be one of the four fixed exit points that are located within the room. Spiders firstly reach the gathering (reception) point before they proceed to their designated exit point. The role of the gathering points is again to prevent the spiders from colliding with each other or other objects when arriving at their final destination. Some spiders might take a detour and move towards the user (Figure 4C) before setting towards the designated gathering point. Spiders that follow this route, move under the user’s chair and then proceed towards their final destination. The probability of a spider to choose a path towards the user’s position depends on the user’s biofeedback and is defined for each spider upon its generation (Figure 4D). Once a spider reaches the exit point, it disappears. An object (e.g., a box) is placed, yet again, in front of each exit point, so the user cannot detect its disappearance location. The user can still notice the spider disappearing behind the object, a fact that gives the simulation a more realistic effect. Spiders follow a straight-line route between each location. These locations were selected, as mentioned before, to prevent the spiders from colliding with one another since the system is not qualified to prevent such accidents.

## Materials and Methods

### Participants

From a pool of 92 applicants, 36 were selected and diagnosed with arachnophobia based on the criteria of the Diagnostic and Statistical Manual of Mental Disorders (5th edition; American Psychiatric Association, 2013). Personal interviews were conducted by an experienced psychiatrist who was trained in the application of the SCID by the author who validated the Structured Clinical Interview (SCID) in Greek populations (Vorvolakos et al., 2006). The 36 participants gave written informed consent. The epidemiological characteristics of the population are presented in Table 1. The 36 participants were divided evenly into two groups, both with equivalent demographics: the Experimental Group which was exposed to the proposed real-time adaptive virtual simulation as described in the System Description section, and the Control Group which was exposed to a pre-recorded static virtual simulation as used by current researches, with the EDA sensor used only as a measurement tool.

**Table 1:** Population table.

Participant genre	Number of participants	Percentage (%)
Gender
Male	18	50
Female	18	50
Age		
18–35	23	64
35–55	8	22
55–72	5	14
Annual economic status		
€14,000.00—€25,000.00	9	25
€25,001.00—€36,000.00	17	47
€36,001.00—€47,000.00	10	28
Marital status		
Married	20	56
Not married	16	44
Academic status		
University degree	24	67
No university degree	12	33
Other medical conditions	
Heart arrhythmia	4	11
Depression	8	22
Stroke*	4	11

*Participants who previously suffered a stroke had recovered and were in very good condition to sit and wear the VR goggles with an EDA sensor.

### Procedure

The procedure of the experiment was implemented at the Biomedical Engineering Laboratory of the National Technical University of Athens, Greece. The study was approved by the Ethics Committee of the National Technical University of Athens with protocol number #37146.

Each of the 36 participants participated in a one-session simulation. The total duration of each session was 20:10 min for a screening test and placement of the equipment, 5 min for the VR simulation, and 5 min for recovery from the VR simulation and a quick screening test to check if there was a complication during the VR simulation. In more detail, in both groups, each participant was given specific instructions about the procedure of the simulation, the duration, and the differences between the States. The participants were informed regarding our study goals, they were asked to sit comfortably on a chair to avoid hand movements to prevent potential movement artifacts during the simulation. Then, the appropriate equipment was placed on each participant. In both groups, the two electrodes of the Arduino Uno Seeed EDA sensor were placed on the left hand, one electrode on the index finger, and the other one on the middle finger. Also, the VR headset was placed on their head. The EDA sensor was recording the Voltage Response difference between the two electrodes. No artifact filter during the recording was applied; removing artifacts is a major issue for the EDA sensor however as we discussed in our previous research (Kritikos et al., 2019d) if the participant remains motionless the artifacts are non-significant. Moreover, to define a common comparison factor for our system, for the specific purposes of this study, we decided to apply Normalization to the Voltage Response measurements, and not extract the phasic and tonic data from the Voltage Response measurements as we did in our previous research (Kritikos et al., 2019d). Finally, at all times a clinician had control of the simulation if something unexpected happened.

**Figure 5:**
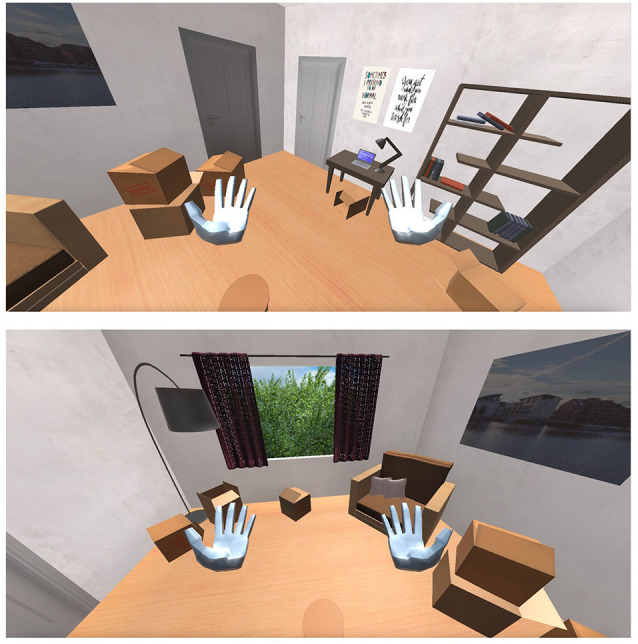
The virtual room in which the patients are exposed.

The VR simulation in both groups has a duration of 5 min and is separated into five phases. During **Phase Zero**, the participant is transferred to a standard virtual office without any phobic stimuli (Figure 5) so that the system can calculate their Relaxed EDA value, which takes 1 min. During this time each participant has the opportunity to explore the virtual office—to feel comfortable with the environment, a short period which proved to be enough for all participants. Once the Relaxed EDA value is defined, **Phase-One** begins. The system is set to the Low-Intensity State (State-1) and monitors the user’s EDA value output for the rest of the minute. Then, **Phase-Two** begins which is the Rest-State (State-0) and has a duration of 1 min. During this phase, we tried to bring the participants’ EDA values back to the Relaxed State values. Next, **Phase-Three** begins. The system is set to High-Intensity State (State-2) and monitors the user’s EDA value output for 1 min. Finally, **Phase-Four** takes place, which is again the Rest State (State-0) and has a duration of 1 min. During this phase, we tried once again to bring the participants’ EDA values back to the Relaxed State values. After this phase, the simulation is terminated.

### Procedural Differences Between Groups

Is important to emphasize that exclusively for the Experimental Group, the EDA sensor output modified the virtual environment according to the aforementioned rules. For the Control Group, the EDA sensor output was used only as a measurement device to calculate and store the Normalized EDA values of the participants, to compare them with the Experimental Group values. In all other aspects, for the Control Group, the duration of the pre-recorded static simulation and the sequence of each Phase are the same as the Experimental Group session. More specifically, in the pre-recorded static simulation, State-0 stays the same, State-1 reflects the stress levels of being mildly anxious, with smaller spiders, moving at a slow speed, bouncing low or not at all, and with few of them moving towards the user, while State-2 reflects the stress levels of being highly anxious, with bigger and more spiders, moving at a relatively faster speed, bouncing relatively higher, with more of them moving towards the user.

## Results and Data Analysis

### Normalized EDA Measurements Over Time and Frequency

**Figure 6:**
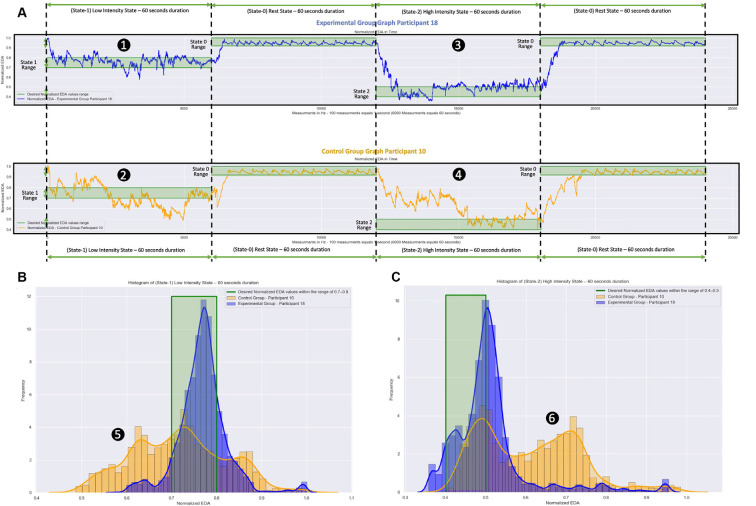
Normalized EDA measurements over time and frequency during the virtual reality (VR) simulation. The blue color graphs are the m easurements of a participant from the Experimental Group. The orange color graphs are the measurements of a participant from the Control Group. The participant from the Experimental Group used a VR simulation which was dynamically changing during the session based on the participant’s EDA measurements. The participant from the Control Group used a VR simulation which was static, that is, the EDA measurements were not affecting the simulation. **(A)** Measurements over Time: **(1)** and **(2)** the Low Intensity State (State-I), which aims to stimulate moderate anxiety, took place during the first minute (0–60 s). Next, during the Rest State (State-0), which aims to bring the participants to their calm state, took place during the second minute (60–120 s). Following that: **(3)** and **(4)** the High Intensity State (State-2), which aims to stimulate high anxiety levels, took place during the third minute (120–180 s). Finally, the Rest State (State-0) during seconds 180–240 follows again during which the participants returned to their calm state. The difference between the measurements of the participant from the Experimental Group and the measurements of the participant from the Control Group is the length of time the participants remained in the appropriately chosen states (desired states). For the participant from the Experimental Group, the EDA measurements show that she/he remained longer within the desired states (green shaded areas), while for the participant from the Control Group, the EDA measurements show that she/he remained for a shorter length of time within the desired states (green shaded area). **(B)** Measurements over frequency during State-I for both participants. **(C)** Measurements over frequency during State-2 for both participants. During the area **(5)**, the participant from the Control Group (orange graphs) had significantly more low-value Normalized EDA measurements compared to the participant from the Experimental Group (blue graphs). During the area **(6)**, the participant from the Control Group (orange graphs) had significantly more high-value Normalized EDA measurements compared to the participant from the Experimental Group (blue graphs).

The reaction to a specific stimulus is usually expressed within 1.6–5.5 s from the moment the stimulus appears and depends on various parameters (reaction latency and recovery time of each participant; Boucsein, 1992; Kappeler-Setz et al., 2013). In Figure 6, the measurements of two participants are presented, one from each group. Those of the 18th participant from the Experimental Group are presented in the blue color graphs. Those of the 10th participant from the Control Group are presented in the orange color graphs.

In Figure 6A, the raw Normalized EDA measurements during the 5-min simulation are presented for both participants. From those two graphs, it can be seen that during State-1 the participant from the Experimental Group (Figure 6A-1) remained inside the desired range of this state for 43.40 s (72.33% of State-1 total duration), while the participant from the Control Group (Figure 6A-2) remained inside the desired range of this state for 20.23 s (33.71% of State-1 total duration). During State-2, the participant from the Experimental Group (Figure 6A-3) remained inside the desired range of this state for 26.62 s (44.36% of State-2 total duration), while the participant from the Control Group (Figure 6A-4) remained inside the desired range of this state for 15.50 s (25.83% of State-2 total duration).

In Figure 6B the frequencies of the Normalized EDA measurements during State-1 for both participants are presented. While the 18th participant from the Experimental Group (blue graph) with Mean = 0.768 and SD = 0.054 had a lot more accumulated Normalized EDA measurements inside the desired range, the 10th participant from the Control Group (orange graph) with Mean = 0.717 and SD = 0.104 had for 26.10 s (43.5% of State-1 total duration) more low-value Normalized EDA measurements in relation to the desired state range (Figure 6B-5).

In Figure 6C the frequencies of the Normalized EDA measurements during State-2 for both participants are presented. While the 18th participant from the Experimental Group (blue graph) with Mean = 0.509 and SD = 0.095 had a lot more accumulated Normalized EDA measurements inside the desired range, the 10th participant from the Control Group (orange graph) with Mean = 0.609 and SD = 0.122 had for 44.45 s (74.08% of the State-2 total duration) more high-value Normalized EDA measurements in relation to the desired state range (Figure 6C-6).

So, the proposed dynamically adaptive VR system caused the 18th participant from the Experimental Group to remain longer in the appropriately chosen stress states, while we can see that the pre-recorded static VR system caused the 10th participant from the Control Group to remain in the appropriately chosen stress states (desired states) for a much shorter length of time. Particularly, the participant from the Control Group with significantly more low-value Normalized EDA measurements during State-1 and with significantly more high-value Normalized EDA measurements during State-2, was stimulated more than it was desired during State-1 (more anxious than it was desired), and was not stimulated enough during State-2 (less anxious than it was desired).

Of course, with modifications to the pre-recorded static VR system, we could achieve for the 10th participant from the Control Group to remain the same time as the 18th participant from the Experimental Group inside the desired states, by making State-1 less anxious and State-2 more anxious. However, in the following section, we will demonstrate that for the same pre-recorded static VR system, different participants from the Control Group reacted differently, some with more anxiety during both states, some with less anxiety during both states, others with more anxiety during State-1 and with less anxiety during State-2, and others with less anxiety during State-1 and with more anxiety during State-2. While the participants from the Experimental Group reacted in similar ways among them to the proposed dynamically adaptive VR system.

### Experimental Group and Control Group Differences

**Figure 7:**
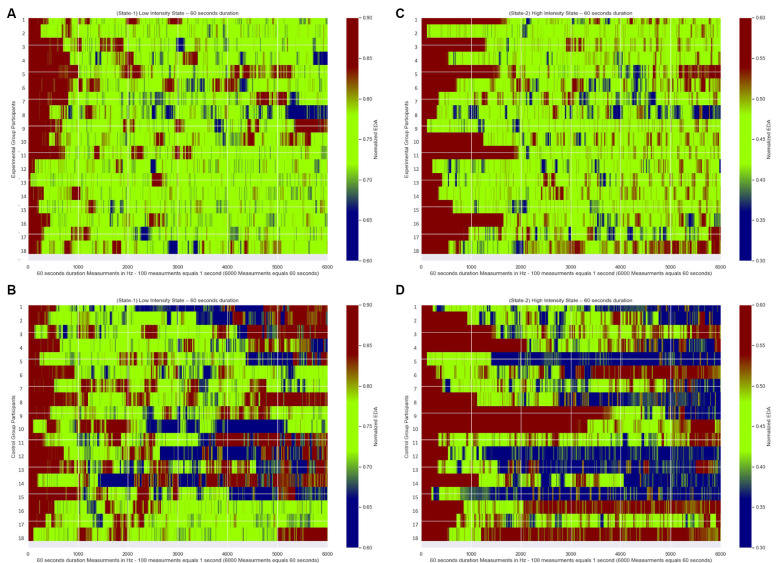
Normalized EDA measurements over time of all participants. The green color corresponds to the desired values of the Normalized EDA measurements for State-1: from 0.7 to 0.8 and for State-2: from 0.4 to 0.5, the red color correspond s to values of the Normalized EDA measurements higher than the desired values for State-I: higher than 0.8 and for State-2: higher than 0.5, the blue color corresponds to values of the Normalized EDA measurements lower than the desired values for State-I: less than 0.7 and for State-2: less than 0.4. **(A)** Measurements during State-I of Experimental Group participants. **(B)** Measurements during State-2 of Experimental Group participants. **(C)** Measurements during State-I of Control Group participants. **(D)** Measurements during State-2 of Control Group participants.

In Figures 7A,B, we present for each of the 18 participants from the Experimental Group their Normalized EDA measurements during State-1 and State-2, respectively. Similarly, in Figures 7C,D, we present for each of the 18 participants from the Control Group their Normalized EDA measurements during State-1 and State-2, respectively. The horizontal axis corresponds to the measurements over time during each State while the vertical axis corresponds to each participant’s Normalized EDA measurements. The color bar corresponds to the values of their Normalized EDA measurements. Particularly, the green color corresponds to the desired values for State-1: from 0.7 to 0.8 and for State-2: from 0.4 to 0.5, the red color corresponds to values higher than the desired value range for State-1: more than 0.8 and for State-2: more than 0.5, the blue color corresponds to values lower than the desired value range for State-1: less than 0.7 and for State-2: less than 0.4.

Moreover, we defined the following variables:

• **State Maintenance Duration**: the length of time in seconds that the Normalized EDA measurements remained within the desired states.
• **Less Anxious Duration**: the length of time that the Normalized EDA measurements had values higher than the desired states, that is, the participants experienced less anxiety than what was expected during the desired states.
• **More Anxious Duration**: the length of time that the Normalized EDA measurements had values lower than the desired states, that is, the participants experienced more anxiety than what was expected during the desired states.
• **State Entrance Time**: the time each participant took to first reach the desired states.
• **Recovery Duration**: the time it took each participant to recover and return to their respective relaxed EDA value at the end of the simulation.

In Tables s 2, 3 we present for each of the 36 participants for both states the values of those variables.

**Table 2:** Experimental group—values per participant during simulation*.

	(State-1) Low-intensity state					(State-2) High-intensity state				
Participant no.	State maintenance duration (s)	Less anxious duration (s)	More anxious duration (s)	State entrance time (s)	Recovery duration (s)	State maintenance duration (s)	Less anxious duration (s)	More anxious duration (s)	State entrance time (s)	Recovery duration (s)
1	44.77	14.01	1.22	4.14	5.09	32.13	24.37	3.50	17.26	5.34
2	53.63	5.19	1.18	2.58	11.56	55.59	3.33	1.08	1.16	1.51
3	43.10	13.55	3.35	7.02	13.02	39.05	20.51	0.44	13.19	7.38
4	38.54	14.20	7.26	8.34	6.28	50.32	7.42	2.26	5.52	3.11
5	33.46	24.10	1.45	10.08	7.25	27.41	29.07	3.52	15.54	10.06
6	35.34	19.46	5.19	9.06	7.25	34.41	20.40	5.19	7.24	14.26
7	40.15	13.50	6.35	8.04	12.16	40.58	12.12	7.30	3.56	15.51
8	33.10	8.42	18.48	4.35	8.34	35.19	7.51	17.3	3.20	13.16
9	39.12	18.34	2.54	5.46	10.58	54.30	3.16	2.54	1.24	3.08
10	42.14	15.37	2.49	4.22	9.06	37.32	20.25	2.43	12.42	5.18
11	45.28	14.26	0.46	7.46	6.36	38.10	21.34	0.56	19.50	11.25
12	53.15	3.50	3.05	1.41	5.02	48.52	5.28	6.20	2.31	9.09
13	56.35	3.45	0.19	0.54	2.56	46.42	11.31	2.27	4.24	4.23
14	52.20	6.51	1.29	3.20	1.51	47.51	12.29	0.20	3.55	7.18
15	51.15	5.33	3.52	2.19	8.03	48.47	7.37	4.16	6.37	8.50
16	49.52	8.12	2.36	4.48	3.41	30.55	24.25	5.20	16.50	13.24
17	47.57	7.18	5.25	3.20	5.57	33.58	17.29	9.13	9.50	18.39
18	43.40	12.33	4.27	1.57	3.94	27.42	29.57	3.01	5.44	6.51
Mean	44.55 (74.25%)	11.49	3.88	4.85	7.05	40.48 (67.46%)	15.38	4.23	8.20	8.72
Std	6.94	5.70	4.04	2.78	3.21	8.72	8.45	3.94	5.83	4.63

* These values are durations measured in seconds. Each state has a total duration of 60 s. State maintenance duration plus less anxious duration plus more anxious duration, equals 60 s, equals the total duration per state.

**Table 3:** Control group—parameters per participant during the simulation*.

	(State-1) Low-intensity state					(State-2) High-intensity state				
Participant no	State maintenance duration (s)	Less anxious duration (s)	More anxious duration (s)	State entrance time (s)	Recovery duration (s)	State maintenance duration (s)	Less anxious duration (s)	More anxious duration (s)	State entrance time (s)	Recovery duration (s)
1	12.04	19.58	28.38	5.54	11.20	24.54	4.41	31.05	2.33	3.57
2	25.25	19.25	15.50	4.06	5.52	22.12	19.56	18.32	9.28	7.40
3	27.18	24.53	8.29	1.31	3.51	22.53	32.23	5.25	15.0	3.39
4	34.19	17.28	8.53	6.40	4.42	12.94	33.50	13.56	21.15	4.40
5	32.26	13.50	14.24	2.32	10.0	12.93	2.54	44.53	1.18	8.13
6	37.02	13.52	9.46	9.31	5.39	15.88	37.11	7.01	3.02	6.55
7	34.56	20.42	5.02	5.18	3.16	30.04	14.44	15.52	4.35	12.19
8	23.33	34.20	2.47	9.25	3.27	13.08	19.56	27.36	11.16	8.06
9	39.53	15.27	5.20	4.50	9.46	8.72	41.19	10.09	38.28	5.42
10	20.33	13.57	26.10	1.10	12.51	15.55	44.45	0.0	33.34	14.36
11	22.29	26.16	11.55	3.51	7.29	35.08	21.53	3.39	3.54	1.55
12	13.51	22.46	24.03	7.19	9.11	5.77	4.23	50.0	5.35	19.54
13	15.29	27.53	17.18	6.30	4.17	9.11	16.59	34.30	3.37	11.44
14	10.03	30.46	19.51	2.07	9.57	17.57	18.25	24.18	11.53	0.57
15	18.44	20.09	21.47	10.51	7.40	4.61	7.29	48.10	2.09	17.21
16	45.37	11.42	3.20	5.29	1.18	10.50	49.50	0.0	8.54	3.05
17	44.23	10.59	5.18	3.53	8.50	32.15	21.56	6.29	7.12	5.33
18	41.11	18.50	1.39	1.10	0.54	5.95	54.05	0.0	5.55	3.50
Mean	27.55 (45.91%)	19.90	12.59	4.91	6.45	16.61 (27.68%)	24.55	18.83	10.34	7.53
Std	11.08	6.46	8.33	2.79	3.39	9.01	15.52	16.46	10.30	5.24

*These values are durations measured in seconds. Each state has a total duration of 60 s. State maintenance duration plus less anxious duration plus more anxious duration, equals 60 s, equals the total duration per state.

#### State Maintenance Duration Differences

In Figures 7A,B, which correspond to the participants’ measurements from the Experimental Group for State-1 and State-2, respectively, there are longer green periods of measurements compared to Figures 7C,D which correspond to the participants’ measurements from the Control Group for State-1 and State-2 respectively. This means that the dynamically adaptive system managed to retain the Normalized EDA measurements of the participants from the Experimental Group very close to or within the desired states for a long time, while the participants from the Control Group, who received a pre-recorded simulation, had Normalized EDA measurements further away from the selected desired states for a longer time.

In more detail, for the statistical analysis, One-Way ANOVA was implemented to compare the State Maintenance Duration values presented in Tables s 2, 3, between Experimental and Control Groups for each state, with α level set at *a* = 0.05. During State-1 there is a significant difference (*F*_(1,18)_ = 28.767, *p* < 0.001) between the Experimental Group participants (with average State Maintenance Duration Mean = 44.55 s, SD = 6.59) and the Control Group participants (with average State Maintenance Duration Mean = 27.55 s, SD = 11.08). During State-2 there is a higher significant difference (*F*_(1,18)_= 61.004, *p* < 0.001) between the Experimental Group participants (with average State Maintenance Duration Mean = 40.48 s, SD = 8.72) and the Control Group participants [with average State Maintenance Duration Mean = 16.61 s (27.68%), SD = 9.01]. This means that during State-1 the proposed dynamically adaptive VR system kept the participants for 74.25% of the time within the appropriately chosen state compared to the pre-recorded static VR system during which participants remained on average for 45.91% of the time within the appropriately chosen state. It also means that during State-2 the proposed dynamically adaptive VR system kept the participants for 67.46% of the time within the appropriately chosen state compared to the pre-recorded static VR system during which participants remained on average for 27.68% of the time within the appropriately chosen state.

#### State Entrance Time, Recovery Duration Differences

Figures 7A,C correspond to the measurements of all participants from both groups during State-1. The first time that participants reached the desired *state* (first green color line) ranged between 0.54 and 10.51 s For the statistical analysis, one-way ANOVA was implemented to compare the State Entrance Time values presented in Tables s 2, 3 between the groups for State-1, with the α level set at *a* = 0.05. By this comparison between Experimental Group participants (with average State Entrance Time Mean = 4.85 s, SD = 2.78) and Control Group participants (with average State Entrance Time Mean = 4.91 s, SD = 2.79) we see that there is no significant difference (*F*_(1,18)_ = 0.00043, *p* < 0.001).

Figures 7B,D correspond to the measurements of all participants from both groups during State-2. The first time that participants reached the desired *state* (first green color line) ranged between 1.16 and 38.28 s For the statistical analysis, one-way ANOVA was implemented to compare the State Entrance Time values presented in Tables s 2, 3 between the groups for State-2, with the α level set at *a* = 0.05. By this comparison between Experimental Group participants (with average State Entrance Time Mean = 8.20 s, SD = 6.00) and Control Group participants (with average State Entrance Time Mean = 10.34 s, SD = 10.30) we see that there is a minor difference (*F*_(1,18)_ = 0.055, *p* < 0.001) yet not a significant difference. This means that the participants from both groups in each State posed no significant difference as regards the time they took to reach the desired state for the first time.

Similarly, comparing the Recovery Duration values, presented in Tables s 2, 3, between the groups for both states, one-way ANOVA was implemented with α level set at *a* = 0.05. During State-1 there is no significant difference (*F*_(1,18)_ = 0.00043, *p* < 0.001) between Experimental Group participants (with average Recovery Duration Mean = 4.85 s, SD = 2.78) and Control Group participants (with average Recovery Duration Mean = 4.91 s, SD = 2.79). During State-2 there is no significant difference (*F*_(1,18)_ = 0.055, *p* < 0.001) between Experimental Group participants (with average Recovery Duration Mean = 8.20 s, SD = 6.00) and Control Group participants (with average Recovery Duration Mean = 8.72 s, SD = 4.77). This means that the participants from both groups in both states posed no difference as regards the length of the recovery time.

#### More, Less Anxious Duration Differences

In Figures 7C,D which correspond to the participants’ measurements from the Control Group there are, both, longer red and blue periods of measurements compared to Figures 7A,B which correspond to the participants’ measurements from the Experimental Group. This means that during the pre-recorded simulation there were participants who were overly exposed (blue periods) to the stimulus and there were participants who were exposed to a stimulus that did not affect them adequately (red periods). As an example, according to Table 3, the first participant demonstrated much more anxiety than it was desired during both states, the 16th participant demonstrated much less anxiety than it was desired during both states, the 10th participant demonstrated much more anxiety than it was desired during State-1 and much less anxiety than it was desired during State-2, while the eighth participant demonstrated much less anxiety than it was desired during State-1 and much more anxiety than it was desired during State-2.

**Figure 8:**
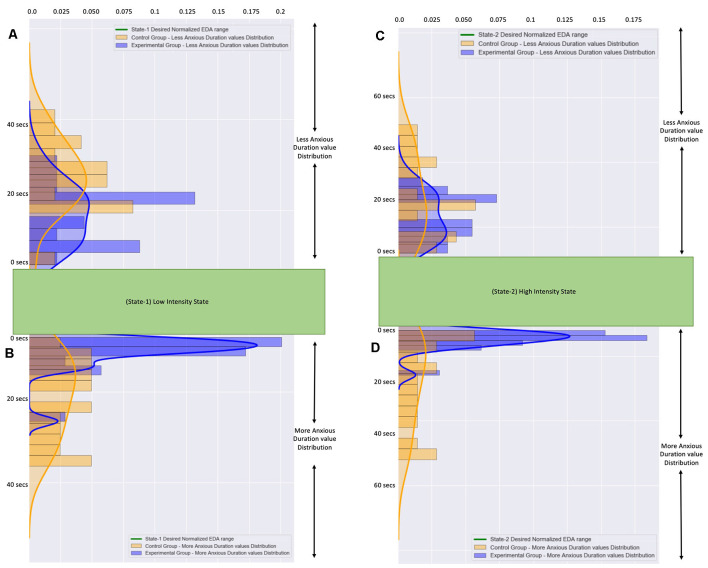
**(A)** The frequency distribution of the less anxious duration values of all participants during State-I. **(B)** The frequency distribution of the more anxious duration valuesof all participants during State-I. **(C)** The frequency distribution of the less anxious duration values of all participants during State-2. **(D)** The frequency distribution of the more anxious duration values of all participants during State-2. The blue color graphs correspond to participants from the Experimental Group. The orange color graphs correspond to participants from the Control Group.The distributions of all values from the Control Group are more scattered.The distributions of all values from the Experimental Group are accumulated close to zero.

In more detail, for the More Anxious Duration values (Figures 8B,D) we observe that the participants from the Control Group (during State-1: Mean = 12.59 s, SD = 8.45 and during State-2: Mean = 18.83 s, SD = 16.46) had much more scattered values compared to the participants from the Experimental Group (during State-1: Mean = 3.88 s, SD = 4.04 and during State-2: Mean = 4.23 s, SD = 4.06) who had their More Anxious Duration values close to zero. The means that there are a few participants from the Control Group who remained much longer with higher anxiety than it was desired, with few of them exceeding 40 s, compared to the participants from the Experimental Group who remained for just a few seconds with higher anxiety than it was desired.

Similarly, for the Less Anxious Duration values (Figures 8A,C) we observe that the participants from the Control Group (during State-1: Mean = 19.90 s, SD = 6.46 and during State-2: Mean = 24.55 s, SD = 15.52) had more scattered values compared to the participants from the Experimental Group (during State-1: Mean = 11.49 s, SD = 5.70 and during State-2: Mean = 15.38 s, SD = 8.45) who had their Less Anxious Duration values close to a few seconds. The means that there are a few participants from the Control Group who remained longer with less anxiety than was desired compared to the participants from the Experimental Group who remained for just a few seconds with higher anxiety than it was desired. It is important to note here that the Means of the Less Anxious Duration values are higher than the Means of the More Anxious Duration values because the Less Anxious Duration value includes the State Entrance Time value.

Therefore, the pre-recorded static VR system provoked, to several participants more exposure and several others less exposure than was desired. In contrast, the proposed dynamically adaptive VR system tried to keep the participants inside the appropriate anxiety level as close as it could for a much longer duration.

#### Differences Between States

Participants from both groups displayed a relatively longer State Entrance Time during State-2 compared to State-1. By calculating Pearson’s correlation coefficient between State-1 and State-2 for the State Entrance Time variable we receive −0.046 as a result, which means a weak linear relationship between State-1 and State-2 State Entrance Time values. For the Experimental Group during State-1, for State Entrance Time variable we have Mean = 4.85, SD = 2.78, and during State-2 for the State Entrance Time variable, we have Mean = 8.20, SD = 5.83. For the Control Group during State-1, for the State Entrance Time variable, we have Mean = 4.91, SD = 2.79, while during State-2 for the State Entrance Time variable we have Mean = 10.34, SD = 10.30. Therefore, participants from both groups need longer to reach for the first time a more intense stimulus.

Similarly, participants from both groups displayed a relatively longer Recovery Duration during State-2 compared to State-1. By calculating Pearson’s correlation coefficient between State-1 and State-2 for the Recovery Duration variable we receive 0.106 as a result, which means a weak linear relationship between State-1 and State-2 Recovery Duration values. For the Experimental Group during State-1, for the State Entrance Time variable, we have Mean = 7.05, SD = 3.21, while during State-2 for the State Entrance Time variable we have Mean = 8.72, SD = 4.63. For the Control Group during State-1, for the State Entrance Time variable, we have Mean = 6.45, SD = 3.39, while during State-2 for the State Entrance Time variable we have Mean = 7.53, SD = 5.24. Therefore, participants from both groups need longer to recover from a more intense stimulus.

### Parameters

To bring the participants from the Experimental Group within the desired states, based on the specific measurements of each one, the dynamically adaptive VR system first calculated the Correction value for every measurement, then it updated the Stimulus Generation Frequency (SGF), Stimulus Jumping Force (SJF), Stimulus Probability Moving Towards User (SPU), Stimulus Size (SS), and Stimulus Velocity (SV) parameters, which affect the VR simulation. And, finally, the virtual environment was changed accordingly.

• If the participants’ Normalized EDA measurements were within the desired range, the Correction values were calculated between −0.1 and 0.0. As the Correction value becomes more negative a stronger stimulus appears. The SGF values were calculated between 120 and 240 fps for State-1, and between 320 and 440 fps for State-2. The SJF values were calculated between 0.035 and 0.049 N. The SPU values were calculated between 0.3265 and 0.4132. The SS values were calculated between 1.20 and 1.357. The SV values were calculated between 0.00471 and 0.00492 m/s.
• If the participants were less anxious than the desired range, the virtual environment had to change to provoke a more intense stimulus. For the system to achieve this, the Correction values were calculated as less than −0.1, so the SGF values, during State-1, were more than 240 fps, and during State-2 more than 440 fps, the SJF values were more than 0.049 N, the SPU values were more than 0.4132, the SS values were more than 1.357, and the SV values were more than 0.00492 m/s.
• If the participants were more anxious than the desired range, the virtual environment had to change to provoke a less intense stimulus. For the system to achieve this, the Correction values were calculated as more than 0.0, so the SGF values, during State-1, were less than 120 fps, and during State-2, they were more than 240 fps, the SJF values were less than 0.035 N, the SPU values were less than 0.3265, the SS values were less than 1.20, and the SV values were less than 0.00471 m/s.

**Figure 9:**
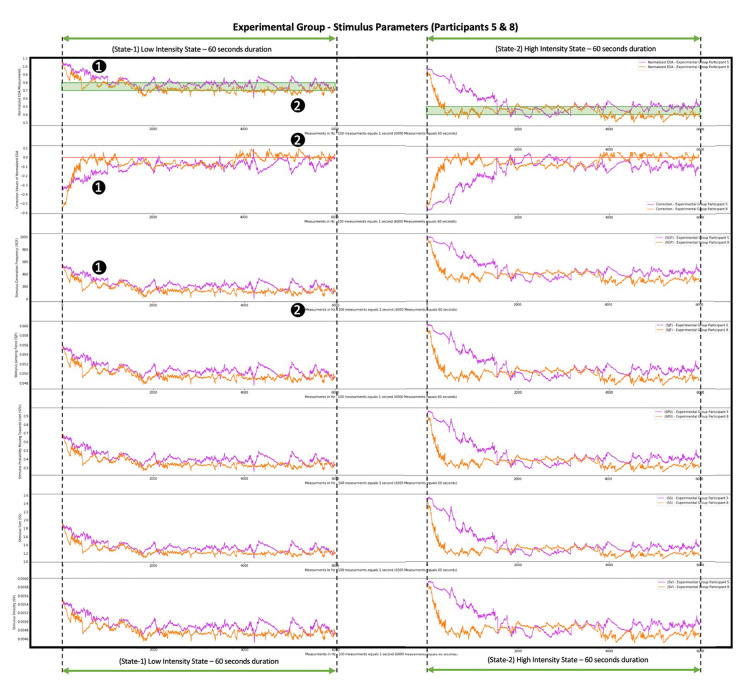
Progression of system correction parameter and Stimulus Generation Frequency (SGF), SJF, SPU, SS, and SV parameters during the simu lation of two participants from the Experimental Group. The graph with the purple color shows the measurements of the fifth participant from the Experimental Group. The graph with the orange color shows the measurements of the eighth participant from the Experimenta l Group. The difference between the two graphs is where they converge: the purple-colored Normalized EDA graph tends to approach values higher than the desired states (area 1), so the fifth participant is harder to stimulate, and a more intense stimulus is needed, thus the correction parameter acquires low values (area 1) and the SGF, SJF, SPU, SS, and SV parameters acquire high values (area 1). The orange-colored Normalized EDA graph tends to approach values lower than the desired states (area 2), so the eighth participant is stimulated more easily, and a less intense stimulus is needed, thus the correction parameter acquires high values (area 2) and the SGF, SJF, SPU, SS, and SV parameters acquire low values (area 2).

By looking at the fifth and eighth participants (Figure 9) we observe the effort performed by the system to bring them and maintain them within the desired anxiety states in a different way. The system recognized that the fifth participant’s Normalized EDA measurements (Figure 9, purple color graph) tended to approach values higher than the desired states, thus the fifth participant was less anxious, was not stimulated as easily, and a more intense stimulus had to be provoked for the participant to approach the desired anxiety states. So, for the fifth participant, this was achieved by increasing the values of the SGF, SJF, SPU, SS, SV parameters. In contrast, the eighth participant’s Normalized EDA measurements (Figure 9, orange color graph) tended to approach values lower than the desired states, thus the eighth participant was more anxious, was stimulated more easily, and a less intense stimulus had to be provoked for the participant to approach the desired anxiety states. So, for the eighth participant, this was achieved by decreasing the values of the SGF, SJF, SPU, SS, SV parameters. For instance, for the fifth participant, on average, the SGF values were 243 fps, and by looking at the purple color graph (Figure 9) in some areas, such as the (1) area, the SGF values exceeded 400 fps, while for the eighth participant, on average, the SGF values were 160 fps, and by looking at the orange color graph (Figure 9) in some areas, such as the (2) area, the SGF values were less than 100 fps, which is an indication of the system’s ability to recognize the difference between participants and adapt the stimulus accordingly.

**Figure 10:**
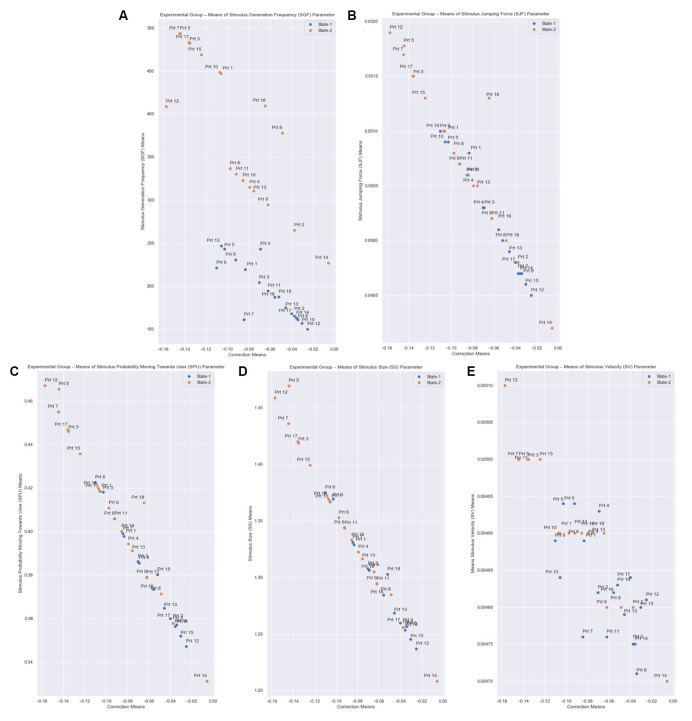
Correction means relative to SGF, SJF, SPU, SS, and SV parameters means of all participants from the Experimental Group for both States. The correction parameter is the subtraction of the State Representative value (Equation 3: desired_eda_normalized) from the Normalized EDA, for every measurement. **(A)** The Stimulus Generation Frequency (SGF). **(B)** Stimulus Jumping Force (SJF). **(C)** Stimulus Probability Moving Towards User (SPU). **(D)** Stimulus Size (SS). **(E)** Stimulus Velocity (SV) parameters are calculated based on the correction parameter (Equations 6–10), for every measurement. Each state has a total duration of 60 s, which equals 6,000 EDA measurements in total per state. Therefore, there is an inverse relationship between the correction value and the parameters.

In Figure 10 we present the means of the SGF, SJF, SPU, SS, and SV parameters relative to the Correction value yielded by the system for each Experimental Group’s participant. We observe that there is an inverse relationship between the Correction value and the parameters. As the Correction value becomes more negative, which means that a stronger stimulus is needed, the SGF, SJF, SPU, SS, and SV parameters take higher values, so that a stronger stimulus appears.

## Discussion and Limitations

### Hypothesis Validation

Fear is recognized in many animal species. Yet there is no consensus in the scientific study of fear. Some argue that “fear” is a psychological construct rather than discoverable through scientific investigation. Studies in both rodents and humans show that there are highly specific brain circuits for fear, although findings from human neuroimaging claim that response to fear is a much broader reaction of the brain (Adolphs, 2013). Nevertheless, the amygdala is a core structure of the brain that seems to orchestrate the reaction towards any fear stimulus (Öhman, 2005). PET works have also revealed a rapid amygdala activation when the person is exposed, very briefly up to 14 ms to pictures of phobic stimuli, such as spiders, and a physiological reaction occurred within 6 s depending on the signal changes (Larson et al., 2006). Thus, a pre-recorded VR system, used for the treatment of phobias cannot facilitate the real-life responses of the brain. In the present study, we proposed and tested a dynamically adaptive VR system that can maintain the patient’s anxiety within a range of desired levels compared to the pre-recorded static VR system simulating a real-life experience. Our results suggest that a system based on a dynamic adaptation of the VR system can be patient’s friendly. As we observed from the participants from the Control Group who used the pre-recorded static VR system, some of them were overly exposed abruptly to the phobic stimuli, and others did not demonstrate the desired electrophysiological response when the stimuli did not affect them adequately. For the specific arachnophobia simulation which we investigated in this study, the extreme reactions during the pre-recorded static VR system did not provoke any unwanted behaviors during the session, but in other forms of simulations, these extreme reactions can provoke significant implications resulting in the delay of the treatment or the patient’s drop out. For example, studies concerning PTSD treatment for veterans (Kramer et al., 2013) mention issues regarding patient safety since the treatment may cause additional stress which may, in turn, lead to them quitting the treatment. Or, for addiction disorders, the uncontrolled VR exposure provocation (Segawa et al., 2020) could increase craving resulting in an inadequate if not harmful treatment. There is evidence that activation of the amygdala by conditioned threat cues is often not observed. This finding could reflect the adaptation of amygdaloid responses over time (Yin et al., 2018). The constantly adaptable environment produced by our VR system incorporates this biological “fitting”. Another important finding is that our method proved efficient to distinguish patients who might need numerous sessions of exposure to the VR phobic stimuli from patients who can be treated in only one session, with obvious financial and treatment outcome advantages. Our findings suggest that it is possible to construct a VR system that has the potential to adjust the psychiatric and neurological treatments according to each patient’s unique characteristics and personality, in real-time, without the need for a system installation. In essence, such systems can be used by clinicians as an additional tool to improve the efficacy of their treatments, as well as to understand better each of their patients’ unique behaviors. This would help them create an actual personalized treatment profile for each participant, based on their unique perception of the stimuli.

Another important factor that we have to consider for a dynamically adaptive VR system is the time it takes the patients to reach the desired state, and the recovery time needed for the patients to get from the desired state to the rest state. In our case, the State Entrance Time required, on average, almost 1/5 of the total treatment time until it reached the desired state for the first time, which is relatively long and affected the results. This led us to conclude that the model by which the state ranges are defined is dependent on how long it takes to reach State-1. Should the time required to reach it is too long, then the desired state range will need to be modified. Another consideration should be the correlation between the State Entrance Time and the Recovery Duration. It was observed that the stronger the stimulus, the more time is needed to reach State-1 for the first time. We feel that this needs to be taken into account when treatment is designed.

Each participant from both groups completed the Presence Questionnaire by Witmer and Singer (1998) at the end of each session. Of the five factors which the Presence Questionnaire assesses, only the questions pertinent to the four relevant factors were included, particularly those pertaining to the factors of Sensory, Involvement, Realism, and Distraction. The questions which are relevant to the Control factor were excluded because in our VR simulation the participants did not interact with virtual objects. They were mainly observing the environment and the stimulus changes during the session. Among the participant responses to the Presence Questionnaire from both the Control and Experimental Groups, no significant difference was observed. We assume that this result occurred because Presence Questionnaire by Witmer and Singer evaluates the presence, immersion, and quality of the virtual environment. In our study, the focus was on the intensity of the stimulus and its variations. Therefore, we recommend that Questionnaires more relevant to stimulus intensity and sensitivity are incorporated in future studies.

In this research, we tried to include as diverse a group of participants as possible, individuals with age, sex, economic, marital, and educational status differences and individuals who have additional illnesses, to test the limits of this proposed dynamically adaptive VR system and how it can adjust to a diverse group of participants. Non-significant differences between different demographic groups were observed. Nevertheless, an additional aim of this system is to provide VR simulations for treating illnesses without knowing the specific medical history and personality traits of participants, to provide controlled and safer treatment sessions. Although the system can provide clinicians with additional information concerning their patients in terms of behaviors, it is designed to function independently to any information of the individual beyond the specific phobia and we recommend further studies take place focusing on demographic differences.

### Limitations: Proposed VR System’s Limitations

Although the feedback results received by the proposed system were promising, there are certain limitations we need to consider.

#### Procedural Limitations

The limited number of different anxiety states and the limited duration of the one-session trial have to be considered. We propose the inclusion and the characterization of alternative orders between states in future related studies to produce an improved and highly responsive version of our system with the ability to update users’ anxiety state each time, regardless of the states induced earlier in the simulation and regardless of the duration of the scenario.

#### Technological Limitations

Our system provided feedback only through a single electrode, the EDA sensor. However, to achieve a more integrated output of the patient’s physiological reactions, dynamic adaptation could be combined with additional electrophysiological sensors that can monitor other human physiological changes (e.g., heart rate). Overall, we strongly believe that a combination of different biosensors applied to similar systems will boost the effectiveness of dynamic adaptation in future studies. It is important to emphasize that several biosensors in the market that provide electrophysiological measurements do not all provide access to the data in real-time. In this research, we used Arduino as the transmitter of the data, which is open access and easy to use device.

Also, another limitation is the quality of the virtual environment. A better definition of objects and an increased number of frames per second can provide a more realistic virtual experience to the users and may yield more accurate findings. By incorporating one or multiple recognition cameras into the experiment, the presence of a virtual user’s body within the virtual room would be achievable, a feature that would make the VR session more interactive and realistic.

#### System Parameterization

All the parameters used in our system, as well as their values, were arbitrarily defined since the aim of this study was to determine whether it is plausible for a system to realize a dynamic adaptation of its virtual environment. Additional studies must be conducted to select the appropriate parameters for the development and implementation of this dynamic adaptation system. Parameters define the way that the system processes the user’s EDA measurements and assists in the correction and adaptation of the virtual stimuli in terms of appearance (size, color, and species) and locomotion (force, velocity, direction, and destination). Moreover, in some cases the parametrization of the system is rather difficult to achieve, so more advanced techniques are required. Techniques such as machine learning algorithms and the addition of neural networks. In this research, we created some parameters for a simple disorder while relying on the literature. However, for complex disorders, the creation of parameters based on observation could not be as effective as we might have wanted them to be. Several researchers have already worked on VR applications with partially dynamic environment generators happening through machine learning (Chen et al., 2017; Padmanaban et al., 2018; Winkler-Schwartz et al., 2019; Alcañiz Raya et al., 2020). Thus, such techniques have the potential of being implemented to improve the parametrization of a dynamically adaptive VR system as proposed in this research.

### Novel Applications in Other Psychiatric and Neurological Conditions

The current use of VR to treat psychiatric and neurological disorders is non-personalized, with predefined, static VR environment scenarios that are not dependent on each patient’s specific needs. Our proposed approach is a personalized, two-way interaction between VR and the patient, with dynamic, non-static VR environment scenarios that can be regulated during the session based on the patient’s specific needs, by measuring their reactions and adapting the VR treatment appropriately. Following our initial objective and after the encouraging results of our trials, we would like to propose novel VR applications for other major neuropsychiatric conditions based on our findings, hoping to potentially provide useful suggestions for further study.

#### Anxiety Disorders

The current VR simulations for Anxiety Disorders are fixed scenarios that initially aim to provoke the specific stressor stimuli, and then patients are instructed to accomplish tasks, gradually, to habituate their anxiety. Based on our approach, by using non-invasive electrophysiological sensors such as those used for heart rate, respiration rate, and electroencephalograms, we measure the anxiety in real-time and adapt the VR scenario based on the specific goal of each treatment. For example, for Social Anxiety Disorder, the VR simulations place the patient inside a virtual room with virtual avatars where the number of virtual avatars can be increased/decreased accordingly or the virtual avatars’ facial expressions can be changed accordingly. Another use could be for Post-Traumatic Stress Disorder for which the scenario would allow the gunfire or the darkness of the VR environment to increase/decrease, or the speed at which the VR environment scenes to change dynamically. In this way, we create a controlled session that can maintain the patient’s anxiety within the desired level/range or can modify accordingly the anxiety levels during the session.

#### Addictions

For Alcohol, drugs, and other addictions, VR has proven to be a useful and safe tool to stimulate addictive scenarios so the patient may gradually confront their addiction. Based on our approach, by using non-invasive electrophysiological sensors such as those used for heart rate, respiration, and electroencephalograms, we can measure the patients’ anxiety in real-time, and adapt the VR scenario according to the specific goal of each treatment. For example, for Alcohol addiction, the patient is placed inside a room with a bottle of alcohol in front of them. Based on the feedback from the sensors, if the patient, just by looking at the bottle, feels anxious, then the environment does not change until the patient is calm and comfortable. If the patient does not feel anxious, then the environment gradually changes, possibly with the appearance of virtual avatars who drink from the bottle.

#### Depression

VR simulations currently used for treating Depression are scenarios that strengthen the patient’s self-confidence by having them execute virtual tasks. Based on our approach which uses non-invasive body-tracking sensors such as accelerometers, motion-tracking cameras, and muscle sensors, we can measure the patient’s behavior and motion during the execution of each virtual task in real-time. More specifically, by measuring how slowly/quickly the patient moves or by measuring how intensely/loosely the patient interacts with the virtual world, based on the feedback from the motion sensors, we create a controlled session that can adapt in real-time the VR scenario based on the specific goal of each treatment session.

#### Stroke Rehabilitation

The current VR simulations for Stroke Rehabilitation are fixed scenarios that aim to mobilize physical activity through gamified scenarios. Based on our approach, by using non-invasive body-tracking sensors, such as accelerometers, motion-tracking cameras, and muscle sensors, we can measure the patient’s movement reactions and adapt the VR scenario based on the specific goal of each treatment. More specifically, by measuring how slowly/quickly the patient moves or by measuring how intensely/loosely they interact with the virtual world, we dynamically create a controlled session and increase or decrease the level of difficulty of each exercise accordingly.

#### Dementias

For Alzheimer’s disease and other dementias, the patients are placed inside the virtual world where they undertake tasks to recall or retrain memories, such as spatial memories and identifying names of common objects or relatives. The currently used VR simulations are scenarios that do not focus on each patient’s specific needs. Even though more personalized VR simulations seem to be required, it is difficult to create VR simulations with the current technology which suits each patient’s specific memories. Nevertheless, more common tasks are more universal and can be dynamically adjusted during VR simulations based on each patient’s specific needs. Such tasks may include language processing skills, naming objects, navigating through a street, or learning to use common objects. For example, inside a virtual room, the patient has to recognize and select specific objects. By using eye-tracking or non-invasive electrophysiological sensors, the VR system can identify during the simulation what objects the patient has difficulty recognizing or interacting with and the system focuses on simulating relative tasks until the patient achieves a relatively satisfying outcome.

#### Psychoses

As regards Schizophrenia and other psychotic disorders, the current VR simulations mainly aim to improve cognitive and social skills as well as job interview skills. They are fixed scenarios during which patients are encouraged to accomplish specific tasks to improve their skills and reduce the burden of cognitive impairment. However, the severity of the psychotic symptoms, condition, and the symptoms, such as delusions, hallucinations, or lack of motivation, make the VR treatment difficult, complex, and potentially unsafe. So, more controlled VR simulations are required. Based on our approach, using eye-tracking sensors inside the VR goggles additionally to non-invasive electrophysiological sensors such as those used for heart rate, respiration and electroencephalograms can provide better control of the treatment adapting in real-time the virtual environment based on the specific goal of each treatment.

## Conclusion

Overall, based on the results obtained from the participants’ responses to all the different simulation states that were controlled by our proposed system, we can conclude that dynamic adaptation is attainable and can be applied in future clinical trials with actual patients. Our system differs from other similar proposals as it gives important emphasis to each person’s unique nature and different perceptions of the same stimuli. The efficacy of the system is confirmed by the results of our study as well since it generated stimuli of a different level to predict and achieve a specific reaction from each participant.

## Data Availability Statement

The raw data supporting the conclusions of this article will be made available by the authors, without undue reservation.

## Ethics Statement

The studies involving human participants were reviewed and approved by the Ethics Committee of the National Technical University of Athens with protocol number #37146. The patients/participants provided their written informed consent to participate in this study. Written informed consent was obtained from the individual(s) for the publication of any potentially identifiable images or data included in this article.

## Author Contributions

IK was involved in the conception and design of the study, in the development of the system, in the data analysis, and in the writing of the original manuscript. GA was involved in the participants’ recruitment, participants’ screening, and manuscript revision. DK was involved in the participants’ recruitment, in the validation of the system, and in the manuscript revision. All authors contributed to the article and approved the submitted version.

## Conflict of Interest

The authors declare that the research was conducted in the absence of any commercial or financial relationships that could be construed as a potential conflict of interest.
